# Application of a needle trap device packed with a MIP@MOF nano-composite for efficient sampling and determination of airborne diazinon pesticide†

**DOI:** 10.1039/d2ra01614a

**Published:** 2022-05-31

**Authors:** Razzagh Rahimpoor, Ali Firoozichahak, Saber Alizadeh, Danial Soleymani-Ghoozhdi, Faeze Mehregan

**Affiliations:** Department of Occupational Health Engineering, Research Center for Health Sciences, School of Health, Larestan University of Medical Sciences Larestan Iran; Department of Occupational Health, Faculty of Health, Social Determinants of Health Research Center, Gonabad University of Medical Science Gonabad Iran a.firoozi@edu.umsha.ac.ir; Department of Chemistry, Bu-Ali-Sina University Hamedan Iran; Student Research Committee, Faculty of Public Health, Kerman University of Medical Sciences Kerman Iran; School of Medicine, Shahrekord University of Medical Sciences Shahrekord Iran

## Abstract

In this research, a novel, selective, and efficient porous adsorbent nano-composite comprising a molecularly imprinted polymer and a metal–organic framework (MIP@MOF) was employed for sampling, extraction and analysis of diazinon from the air by a needle trap device (NTD), for the first time. The synthesized MIP@MOF sorbent was characterized by the FT-IR, XRD, FE-SEM, TEM, and EDS techniques. Then, the effective parameters of the sampling (temperature and humidity) and desorption (time and temperature) process were optimized by response surface methodology (RSM). The optimum values of temperature and humidity of the sampling chamber were estimated to be 20 °C and 25.0%, respectively. Also, the highest response during the analyte desorption was obtained at 262 °C and 4.5 minutes. For more details, the performance of the MIP@MOF:NTD method was evaluated by determination of important parameters such as repeatability, reproducibility, the limit of detection (LOD), and the limit of quantification (LOQ), and then compared with the NIOSH 5600 standard method. The values of LOD and LOQ for the targeted analyte were determined to be 0.02 and 0.1 μg m^−3^, respectively. Also, the repeatability and reproducibility of the proposed method were obtained in the range of (3.9–5.1)% and (5.1–6.4)%, respectively, which proved the acceptable precision of the method. Furthermore, the results of this study exhibited a high correlation coefficient (*R*^2^ = 0.9781) between the proposed method and the recommended NIOSH method. Finally, the proposed procedure was utilized for sampling and determination of the airborne diazinon in real conditions. These results indicated that the proposed MIP@MOF:NTD method can be employed as a fast, simple, environmentally friendly, selective, and effective procedure for sampling and determining diazinon in air.

## Abstract 2

Schematic diagram of the sampling chamber.

## Graphical abstract

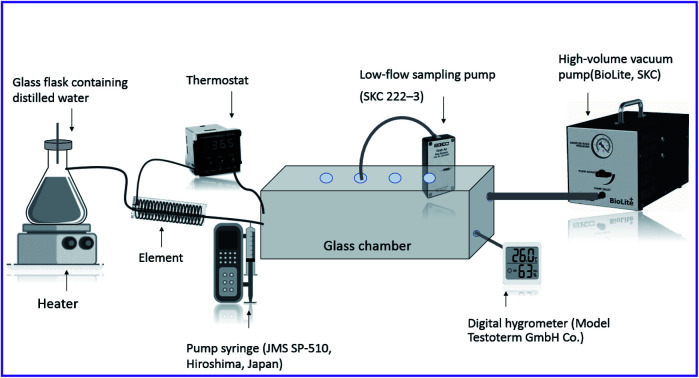

## 1. Introduction

Pesticides as dangerous chemical materials are widely used to control pests and weeds due to their high biological activity. Sometimes, the long-term durability of these poisonous materials in the environment can be a harmful issue for human health and the environmental eco-system.^1,2^ Organophosphate pesticides such as diazinon are some of the most well-known organic pollutants.^3^ Diazinon is one of the most widely used pesticides due to its efficiency and cost-effectiveness.^4^ The complications and symptoms of diazinon poisoning are very diverse and can be occurred at different intervals after exposure, including damage to the nervous system, liver, and kidneys.^5–7^ For this reason, diazinon has been classified in Group 2A by the International Agency for Research on Cancer (IARC).^8^ Most pesticides can be found in the air with low concentrations. So, sampling and determination of them require selective, sensitive, and reliable methods.^9^ The National Institute for Occupational Safety and Health (NIOSH 5600) has proposed a sorbent tube containing XAD-2, followed by gas chromatography for sampling and analysis of organophosphate pesticides in the air.^10,11^ But, the various disadvantages such as multi-step sample preparation and use of the organic solvents for analyte extraction because of the expensive, toxic and carcinogenic reasons should be considered.^11^

In recent years, there has been a growing interest in developing a rapid, one-step, simpler, solvent-free, and cost-effective method for the determination of diazinon.^12,13^ Solid-phase microextraction (SPME), developed by Pawliszyn and Arthur in the late 1990s, is a microextraction technique with a thin layer of adsorbent coated onto a fibre. To sample with SPME, the fiber should be exposed to the sample and the targeted analytes are divided between the coating and the sample matrix.^14^ Despite the advantages such as free-solvent, high sensitivity, and simple implementation,^15^ SPME has disadvantages such as the limited variety of commercial fibers, short life and fiber fragility, as well as limited adsorption capacity.^16,17^ The needle trap device (NTD) which was introduced by Pawliszyn *et al.* in 2001,^18^ is a new needle-based microextraction method for air sampling. In the NTD technique, a certain length of a stainless steel needle is packed with different types of commercial or new and synthesized adsorbents.^19–23^ Compared to the SPME method, the extraction of analytes can be done without relying on the emission principle. Also, the adsorption capacity can be increased as more adsorbent is packed into the needle.

It should be noted that NTD as a flexible method can be used with different adsorbents as a packaging agent^24–26^ Metal–Organic Frameworks (MOFs) are a new class of porous materials that consist of metal ions or clusters and organic ligands as binders and the diversity of this type of adsorbents is due to the diversity in the type of ligand and metal.^27–29^ This group of adsorbents have unique properties compared to commercial adsorbents such as high porosity, excellent thermal stability, high adsorption level as well as adjustable pore structure and size.^30–32^ For this reason, it has been employed in various fields such as drug delivery, gas storage, sensors, catalysts, electrocatalyst, supercapacitor, and sampling of various analytes.^33–39^

Molecularly imprinted polymers (MIPs) are synthetic polymers with specified functions for selectively detection of targeted analytes. These adsorbents which are synthesized using a three-dimensional polymer network according to the shape and size of the target analyte, enable the specific adsorption and recycling of the desired analytes from a variety of samples.^40^ Due to their easy synthesis, low cost, reusability, high efficiency, high stability, and resistance in different conditions, MIPs have been used in many fields, especially as selective adsorbents for SPE, chromatographic separation, and sensors.^41–43^ However, in traditional MIPs, their flexible skeletons lead to disadvantages such as incomplete removal of templates and irregular particle size and shape, slow mass transfer, and low adsorption capacity.^44^

The reported documents about the MIP as an adsorbent in NTD are limited. Boying Yue *et al.* used the NTD:MIP method for the extraction of polycyclic aromatic hydrocarbon in water as an effective and efficient method.^45^ Also, the method of inside-needle adsorption trap (INAT) with MIP was used to determine amphetamine and methamphetamine followed by GC-FID.^46^

In recent years, various supports have been used to stabilize monomers and template molecules, such as quantum dots, Fe_3_O_4_ nanoparticles, gold nanoparticles, surfactants, and carbon nanotubes.^47,48^ Compared to these materials, MOFs are a good choice as a support item due to their high surface area, high porosity, and thermal and chemical stability.^49^ So far, different structures of MOFs have been employed to support of MIPs in various studies.^50–52^ To the best of our knowledge, there is no document for micro-extraction and determination of diazinon in the air by NTD packed with the MIP@MOF nano-composite.

According to this background, in this study, MIP@MOF nano-composite was employed as a novel, selective, and efficient porous adsorbent for sampling, extraction and analysis of diazinon from the air by a needle trap device (NTD), for the first time. In this way, optimizing important conditions of sampling and desorption was performed by a statistical response method Response Surface Methodology (RSM) software. Furthermore, to validate the proposed method various analytical parameters such as the limit of quantification (LOQ), Limit of Detection (LOD), storage stability, breakthrough volume, and carryover were evaluated. Finally, the performance of the MOF@MIP:NTD technique was surveyed in the real workplace samples and compared with the standard method of NIOSH 5600.

## 2. Materials and methods

### 2.1 Chemical materials

To synthesize of MIP@MOF nano-composite as a sorbent, ethylene glycol demethacrylate (EGDMA; 98.0%), 2-azobisisobutyronitrile (AIBN; 98.0%), terephthalic acid (H_2_BDC; 97.0%), ferric chloride hexahydrate (FeCl_3_·6H_2_O), dimethylformamide (DMF; 99.8%), methacrylate acid (MA; 99%), acetonitrile (99.8%), methanol (99.8%) and acetic acid, toluene (99.0%), and acetone (99.0%) were purchased from Sigma Aldrich and used without any purification. Also, diazinon (60.0%) was purchased from Merck Co (Darmstadt, Germany). Nitrogen with high purity (99.99%) was obtained from Roham Co (Tehran, Iran). XAD-2 sorbent tubes (270 : 140 weight ratio) were purchased from SKC.

### 2.2 Synthesis of MIL-101(Fe)

MIL-101(Fe) was synthesized based on a previously reported procedure with some modifications.^53^ Briefly, 1.03 g of H_2_BDC and 3.38 g of FeCl_3_·6H_2_O were mixed at the 50.0 mL of DMF and were shaken gently for 20 min. Then, the solution was placed in a Teflon autoclave at 115 °C for 20 hours. Eventually, the obtained powder was separated by centrifugation. Then, it was washed 5 times with water (20.0 mL) and 5 times with ethanol (15.0 mL). The prepared MIL-101(Fe) was dried at room temperature and activated at 100 °C.

### 2.3 Synthesize of MIP@MOF core–shell

MIP@MOF core–shell was synthesized based on a previously reported procedure with some modifications.^54^ Initially, 126.0 mg of diazinon as a template was combined with the synthesized MIL-101(Fe) (20.0 mg), methacrylic acid (functional monomer: 142.0 mg), methanol (9.43 mL), and acetonitrile (28.3 mL) to the preparation of the MIP@MOF nanocomposite. The resulting mixture was stirred vigorously for 60 minutes. Then, 1.88 mL of EGDMA and 20.0 mg of AIBN were added to the reaction vessel. The prepared mixture was sonicated for 60 minutes at 25 °C. The polymerization process was performed by placing the reaction vessel in an oil bath at 60 °C and stirring at 250 rpm for 24 h under a nitrogen atmosphere. After the polymerization process, the prepared polymer was worked up by methanol : acetic acid (1 : 9) composition in a Soxhlet apparatus to the removal of the diazinon template from the polymer structure. Finally, MIP@MOF nano-composite was dried using a vacuum oven at 65 °C for 10 h.

### 2.4 Instruments and pilot study

**Fig. 1:**
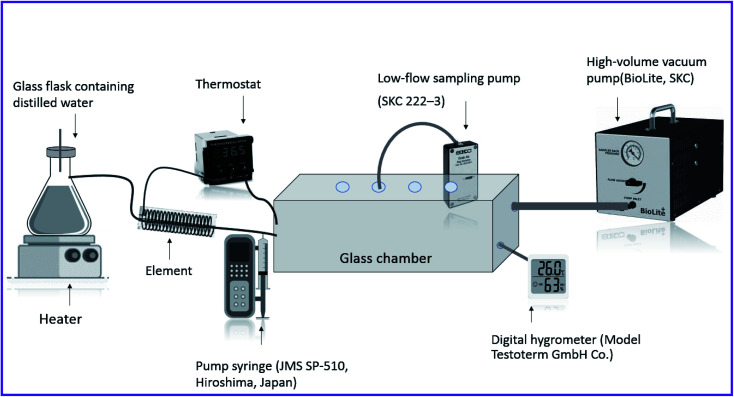
Schematic diagram of the sampling chamber.

A homemade atmospheric chamber was used to create different concentrations of diazinon in the air as shown in Fig. 1. This glass chamber with dimensions of 15.0 × 20.0 × 30.0 cm including NTD and NIOSH method sampling, temperature and humidity monitoring sensors. Diazinon was injected purely in the pumping syringe (SP-510, JMS) and different concentrations of diazinon were created by adjusting the injection rate inside the chamber. The humidity of the chamber was controlled by using an Erlenmeyer on the hot plate (PT100-FALC) and was measured by a digital hygrometer (Testoterm-GmbH). The temperature of the chamber was also adjusted using an element and a thermostat connected to a heat sensor (SUN15-TI). The produced diazinon vapours in the chamber were diluted by a high-volume vacuum pump (BioLite Sampling pump, SKC) to reach the desired concentration. A low-flow sampling pump (222–3, SKC) was used for air sampling by NTD (spinal needle, 22-gauge Tokyo, Japan). Also, a sorbent tube (XAD-2, 270 mg/140 mg) for sampling by the standard method (NIOSH 5600) was purchased from SKC (USA). It should be noted that because of the possibility of adsorption of analytes by the wall of the chamber; sampling of analytes was performed after 30 minutes of the air passage.

The determination of the diazinon compound was performed by a Varian CP-3800 gas chromatography system with a capillary column Chrompack CP7860 column (CP-Sil 5 CB, 30 m × 0.25 mm × 0.33 μm) followed by a flame ionization detector (GC-FID). The high pure nitrogen gases (99.999%) with a flow rate of 4.0 mL min^−1^ were used as carrier gas. The injection port and FID temperature were maintained at 200–300 °C and 280 °C, respectively. The X-ray diffraction (XRD) analysis was done by a Bragg–Brentano XRD apparatus (2*θ*, 5–80° geometry; Cu Kα: model APD 2000; Italy). The Field-Emission Scanning Electron Microscopy (FE-SEM) analysis of prepared MIP@MOF nanocomposite was recorded by TESCAN MIRA3 instrument. Also, the Infra-red absorption analysis was carried out by an ABB FTLA 2000 Fourier transform infrared spectrometer (400–4000 cm^−1^).

### 2.5 GC analysis

All of the samples were analyzed by gas chromatography (Varian CP-3800) with Flame Ionization Detector (FID) equipped with a CP7462 column (30 m × 0.25 mm) capillary column. The temperature programming of the column was started at 60 °C and then raised to 200 °C with a rate of 10 °C min^−1^ for 1.0 min. Then, it was increased to 280 °C with a rate of 10 °C min^−1^ and kept for 2 min. Finally, the FID temperature and injection port were set at 280 °C and 200–290 °C, respectively.

### 2.6 MIP@MOF:NTD preparation

In this study, a spinal needle (22 G, 90.0 mm × 0.71 mm. Kosan, Japan-Tokyo) was packed with MIP@MOF adsorbent as the NTD. In this way, 1.0 mg of adsorbent was mixed with 1.0 mg crushed glass (to preventing of blockage) and was packed in the needle. To the protection of the absorber, both sides of the needle were blocked by glass wool. It should be noted initial tests showed that the crushed glass had not any adsorption. Finally, the airflow through the needle trap was measured using a soap bubble calibration circuit.

### 2.7 Response surface methodology (RSM)

RSM was introduced in 1951 by Box and Wilson for the first time. This method is a set of mathematical and statistical techniques based on linear or square polynomial functions to construct experimental models.^55^ This method can effectively optimize the levels of variables that affect the performance of the NTD method. In this study, the response surface methodology based on a central composite design (CCD) was used for the investigation of effective parameters such as desorption temperature and time, sampling temperature and humidity. The CCD design makes it possible to study linear effects, binary interactions, and quadratic effects of variables.

### 2.8 Optimization of effective parameters

#### 2.8.1 Desorption parameters

The desorption time and temperature are important parameters that affect the performance of the NTD technique. The MIP@MOF adsorbent was used in the NTD technique for the first time, therefore, it is very important to determine the optimal factors affecting the adsorption of diazinon. Response surface methodology (RMS) was utilized for the optimization of these parameters. In the present study, the desorption temperature and time were investigated in the range of 200–290 °C and 1–6 minutes respectively, at a concentration of 0.01 mg m^−3^.

#### 2.8.2 Sampling parameters

One of the most important parameters that can be affected the NTD performance and other extraction methods, is the temperature and relative humidity of the sampling site. In this study, these parameters were optimized by utilizing Design expert software (version 10) and the central composite method. For this purpose, sampling temperature and relative humidity were investigated in the range of 20–60 °C, and 25.0 to 70.0%, respectively.

### 2.9 Method validation

For more details, the performance of the MIP@MOF:NTD method was evaluated by evaluation of important parameters such as repeatability, reproducibility, the limit of detection (LOD), limit of quantification (LOQ) at the experimental method and then compared with NIOSH 5600 standard method. Thus, the concentration of diazinon in the standard chamber was gradually decreased diluted up to signal-to-noise ratio (3 and 10) with 5 replicates. Repeatability and reproducibility of MIP@MOF:NTD was expressed using relative standard deviation (RSD%). To determine repeatability, sampling was performed by 5 replicates at different concentrations (0.002–0.03 mg m^−3^). Also, the reproducibility of the method was evaluated by sampling at a concentration of 0.01 mg m^−3^ using three similar needles (in terms of airflow and adsorbent).

### 2.10 Breakthrough volume (BTV)

The BTV for a special adsorbent depends on the parameters such as the type of adsorbent, analyte affinity, the amount of adsorbent (adsorbent length), and analyte concentration of the needle.^56^ To determine BTV, two similar NTDs with equal amounts of adsorbent were connected in series (NTD-1 at the front and NTD-2 at the back). Sampling was performed at different temperatures with a concentration of 0.01 mg m^−3^. NTD-2 was then injected into the GC for the detection of analytes. Finally, BTV was calculated in terms of mL.

### 2.11 Carryover effect

Carrying over is a problem with analyte desorption where a significant amount of analyte remains on the adsorbent after the desorption step. The residual analyte can cause errors in subsequent measurements.^57^ For this reason, the desorption time and temperature must be selected correctly to minimize the amount of carryover. In this study, other than in optimal temperature and desorption time, carryover was also investigated at other times.

### 2.12 Storage time

To evaluate the storage time of diazinon in MIP@MOF:NTD, sampling was performed under optimal conditions at a concentration of 0.01 mg m^−3^. After sampling, both sides of the NTDs were sealed and the samples were stored at 25 and 4 °C for 1 to 60 days to evaluate the storage capability.

### 2.13 Measurements in a real environment

To investigate the MIP@MOF:NTD performance in a real workplace, the efficiency of the proposed method and NIOSH 5600 method (XAD-2 sorbent tube), was tested. For this goal, 10 farmers who sprayed their crops with diazinon was selected as real sample. Finally, the obtained results from NTD method were compared with the standard method (NIOSH 5600).

## 3. Results and discussion

### 3.1 Characterization of the MIP@MOF

For more details, FT-IR, XRD, FE-SEM, and HR-TEM analyses were performed to the evaluation of the functional groups, crystallinity, and morphology of the synthesized adsorbents.

**Fig. 2:**
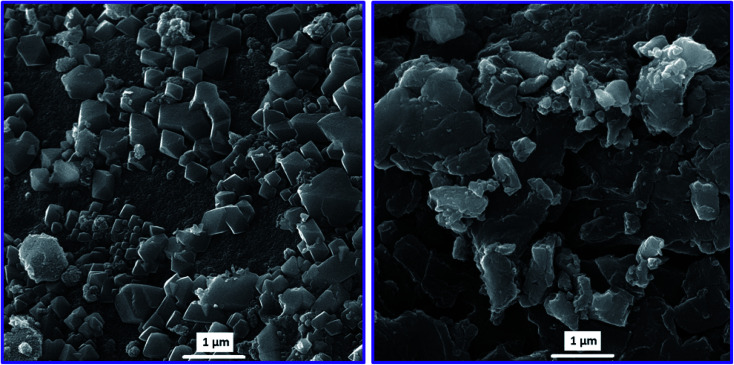
FE-SEM images of MIL-101(Fe) (left) and MIP@MOF nanocomposite (right).

The FE-SEM images were recorded for a surface morphological survey of synthesized MIL-101(Fe) and MIP@MOF nanocomposite crystals. The FE-SEM image of MIL-101(Fe) (Fig. 2 left) indicates uniform polyhedral structures with dimensions between 370 to 420 nm. The FE-SEM image of the MIP@MOF nanocomposite (Fig. 2 right) clearly shows that the polyhedral structure of MIP@MOF crystals was covered and enclosed by the MIP films. According to Fig. 2, MIP@MOF has a core–shell structure in which the polyhedral MIL-101(Fe) cores are covered by the MIP shells. So, the obtained images can be proved the successful synthesis of MIP@MOF core–shell structures.

**Fig. 3:**
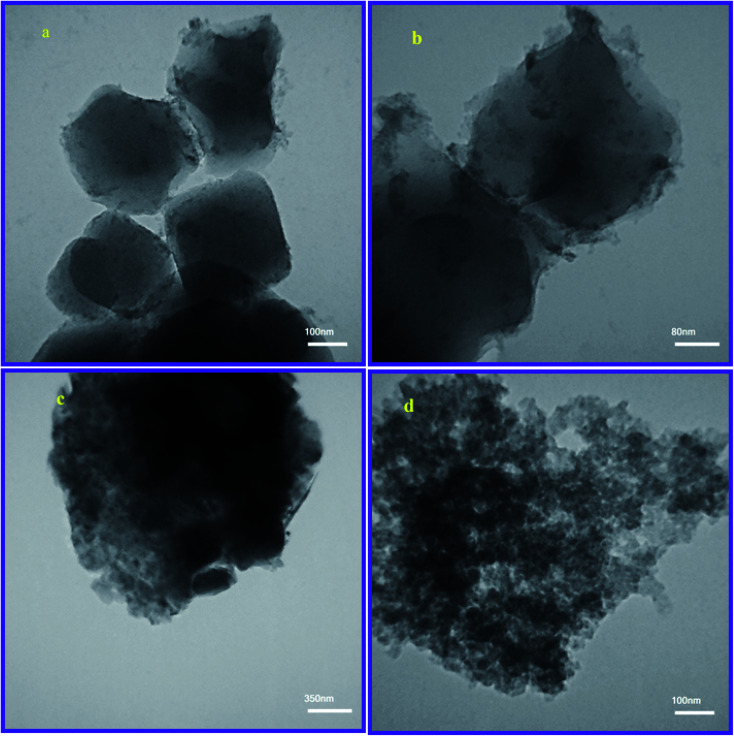
HR-TEM image of MIL-101(Fe) (a and b) and MIP@MOF (c and d).

Furthermore, the HR-TEM analysis was performed for a thin-film investigation of the prepared sorbents. Fig. 3a and b show the TEM images of MIL-101(Fe) crystals. According to the images, the thin films of MIL-101(Fe) crystals shows the uniform and four square structures in which the dimension of each face is 200–300 nm. Also, Fig. 3c and d shows the TEM images of MIP@MOF nano-composite. Based on the images, the darker and thicker regions of thin films with an amorphous appearance can be approved for the coverage of MIL-101(Fe) crystals by MIP layers like a shell of cores.

FTIR analysis was performed to evaluate the functional groups of MIL-101(Fe) and MIP@MOF structures. The FT-IR spectra of MIL-101(Fe) and MIP@MOF nanocomposite are shown in Fig. S1(a and b).† The bands at 1580.84 and 1654.55 cm^−1^ were attributed to the C=O bond in the carboxylates. Also, the bands at 1381.87 and 1418.79 cm^−1^ indicate C=C vibrating bands that are consistent with other studies.^54,58–60^

Also, the XRD analysis was performed for the investigation of the purity and crystallinity structure of the synthesized MIL@MOF nanocomposite. The appeared featured diffraction peaks at the recorded pattern proved the purity and crystallinity structure of the synthesized MIL@MOF nanocomposite which is compatible with the previously reported document.^54,58–60^ According to Fig. S2(a),† the XRD pattern of MIP indicates approximately amorphous structures, which is consistent with the recorded FE-SEM images. Also, Fig. S2(b)† illustrates the simultaneous presence of the main diffraction peaks of MIL-101(Fe) crystals (2*θ* = 7.0°, 10.0°, 15.0°, 26.2°) and the main diffraction peaks of MIP (18.05° and 23.0°). This pattern illustrates approves the successful preparation of MIP@MOF core–shell structures. For more details, the XRD patterns of MIL-101(Fe) and MIP are also presented for comparison in Fig. S2† which confirms the successful synthesis of MIP@MOF core–shell structures.

In the following, the BET analysis was performed to an evaluation of the MIL-101(Fe), MIP, and MIP@MOF specific surface area. According to the BJH diagram, the specific surface area of MIL-101(Fe), MIP, and MIP@MOF structures were 1981.0, 683.0, and 375.0 m^2^ g^−1^, respectively. Also, the total pore volume of MIL-101(Fe), MIP, and MIP@MOF structure was determined to be 1.18, 0.117, and 0.125 cm^3^ g^−1^, respectively. The decreasing of the specific surface area and total pore volume of MIP@MOF nanocomposite indicates that MIP polymer is well established in the structure of MOF.^54,58–60^

### 3.2 Desorption parameters

**Fig. 4:**
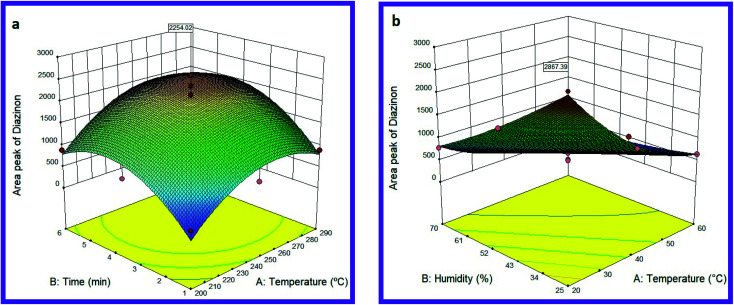
Effect of desorption (a) and sampling (b) variables on the efficiency of MIP@MOF:NTD in the determination of diazinon.

In the present study, the effect of different desorption temperatures (200, 230, 250, 270, and 290 °C) and different desorption times (1, 2, 3, 4, 5 and 6 min) were evaluated on the MIP@MOF:NTD performance. Fig. 4 shows the optimal temperature–time interaction for diazinon desorption. The results exhibited that the highest response can be observed at 262 °C and 4.5 minutes (shown in Table S1†). The validation and regression coefficient model was obtained by Design-Expert software and analysis of variance (ANOVA). The values of *R*^2^, adjusted *R*^2^, and coefficient of variation (CV) are shown in Table S2.† The results showed that the response was dependent on the input variables (*R*^2^ > 0.8), the closeness of *R*^2^, and *R*^2^ values to 1.00 indicated the compatibility of the quadratic model for diazinon desorption. The lack-of-fit value (*P* > 0.05) also showed that the temperature and time parameters and their interactions are very effective on adsorption efficiency. The results of this study are consistent with other similar studies. For example, Jafari *et al.* have reported polypyrrole/montmorillonite as a sorbent in the SPME method for the extraction of diazinon and fenthion with a desorption temperature and time of 260 °C and 4 minutes, respectively.^61^ Also, Amini *et al.* have reported PAN/Ni-MOF as a fiber coating in the SPME method for the extraction of diazinon by GC with a desorption temperature of 200 °C.^62^ In one study, Ahmadkhaniha used single-walled carbon nanotubes coating as a sorbent in SPME for the determination of parathion, malathion, diazinon, and pirimiphos-methyl, which reported desorption temperature and time of 230 °C and 4 minutes, respectively.^63^

### 3.3 Sampling parameters

The effects of relative humidity and sampling temperature on the MIP@MOF:NTD performance for diazinon are shown in Fig. 4. The best performance was obtained at 20 °C and 25.0% relative humidity (Table S1†). As shown in Fig. 4, the efficiency of the proposed method leads toward the lower amounts along with the increase in temperature and humidity.

The optimum values of humidity and sampling temperature were estimated by RSM (Quadratic model). The modelling results (including *R*^2^, adj. *R*^2^, CV and lack-of-fit) are presented in Table S3.† As can be seen, in the quadratic model, the values of *R*^2^, *p*-values and Lack of Fit were determined to be 0.97, <0.0001, and 0.6069 respectively, which indicates the appropriate response of the model. According to the results, the performance of MIP@MOF:NTD will be better at lower temperatures and humidity. Competition of targeted analyte with water vapour during of adsorption process can be a reason for reducing the performance of proposed methods at the higher temperature and humidity.^64^ Moreover, increasing the sampling temperature increases the molecular movement and thus reduces the efficiency of surface adsorbents.

### 3.4 Carryover effect

In general, carryover depends on the temperature and desorption time. In this study, carryover was not observed at the optimum point (desorption time of 4.5 minutes and desorption temperature of 262 °C). But for more details, it was investigated for other desorption times (Table 1). As can be seen, the maximum memory effect is related to the adsorption time of one minute.

**Table 1:** Carryover effect of MIP@MOF:NTD at different desorption times^a^

Carryover effect						
Time (min)	1	2	3	4	5	6
Carryover (%)	0.39	0.26	0.10	ND	ND	ND

a ND: not detected.

### 3.5 Breakthrough volume (BTV) investigation

To evaluation of BTV, the concentration of analyte in the sampling chamber was adjusted to be 0.01 mg m^−3^. As shown in Table 2, the breakthrough volume leads to lower values by increasing the sampling temperature. The lowest BTV was detected at 60 °C (1100.0 mL). This phenomenon can be justified by the correlation of temperature and vapour pressure of each compound. So that, the vapour pressure of the analyte can be increased along with the increase of temperature. Increasing the vapour pressure reduces the adsorption tendency of the adsorbent, which leads to the limitation of the adsorption capacity. Therefore, BTV leads to lower volumes at constant concentrations.^64^

**Table 2:** The breakthrough volume of NTD:MOF@MIP for diazinon at five different temperatures

Breakthrough volume					
Temperature (°C)	20	30	40	50	60
Breakthrough volume (mL)	2720.0	2630.0	2410.0	1940.0	1100.0

### 3.6 Method validation

#### 3.6.1 Repeatability and reproducibility

Repeatability and reproducibility were determined in the concentration of 0.002–0.03 and 0.01 mg m^−3^, respectively (under optimal conditions). The results of repeatability and reproducibility based on RSD% were obtained in the range of (3.9–5.1)% and (5.1–6.4)%, respectively. The repeatability of the standard NIOSH method (5600) for sampling and analysis of diazinon was reported to be 16.0%, which is comparable to the results of the present study. Also, this parameter was estimated in the range of (3.9–8.1)% for diazinon by Ramezani *et al.* research.^65^ Also, the repeatability was reported in the range of (4.0–10.1)% by Amini *et al.* group.^62^ As shown in Table 3, there was no significant difference in reproducibility and repeatability of the selected samples which indicates the appropriate precision of the proposed method.

**Table 3:** Reproducibility and repeatability of MIP@MOF:NTD for sampling and analysis of diazinon

RSD%								
Parameters	RSD% for a NTD at different concentrations (mg m^−3^)					RSD% for different NTDs at a constant concentration (0.01 mg m^−3^)		
Concentration/NTD	0.002	0.006	0.01	0.02	0.03	NTD1	NTD2	NTD3
RSD%	3.9	4.6	4.8	5.1	4.2	6.4	5.1	5.9

#### 3.6.2 LOD and LOQ

In the following, the MIP@MOF:NTD method was employed for the determination of LOD and LOQ parameters. The LOD and LOQ parameters were calculated to be 0.02 and 0.1 μg m^−3^, respectively which was lower than the standard method of NIOSH and other studies. For example, the LOD value of the NIOSH method for the determination of diazinon in the air is reported to be 0.04 μg m^−3^.^10^ Also, the LOD value was reported to be 0.02 ng mL^−1^ in the reported study by Russo *et al.* for the determination of diazinon by XAD-2 tube in the air.^66^ Also, in another study, LOD and LOQ values for sampling organophosphate pesticides from the air were reported to be 0.07 and 0.2 μg m^−3^, respectively.^9^ The proposed method in this study was compared with the other reported methods which are shown in Table 4.

**Table 4:** Comparison of MIP@MOF:NTD with other techniques for determination of diazinon

Technique	Determination	Matrix	LOD	LOQ	RSD (%)	Ref.
NIOSH 5600	GC-FPD	Air	0.04 μg sample	—	16.0	10
DI-SPME	GC-CD-IMS	Water, apple, vegetable	0.02 ng mL^−1^	—	5.1–9.4	61
HS-SPME	CD-IMS	Aqueous media	0.3 ng mL^−1^	0.1 ng mL^−1^	4.0–10.1	62
HS-SPME	GC-MS	Dried medicinal plants	0.3 ng g^−1^	1.5 ng g^−1^	6.7–10.8	63
HS-SPME	GC-MS	Water–fruit juices	0.02 ng mL^−1^	0.07 ng mL^−1^	3.9–8.1	65
HS-SPME	GC-NPD	Water–soil	0.005 ng mL^−1^	0.015 ng mL^−1^	6.0–8.1	67
Smart SPME	GC-FID	Wheat	4.0 μg kg^−1^	—	5.6–13.1	68
SPE cartridges	GC-NPD	Air	0.02 ng mL^−1^	—	8.2	66
Solid sorbent tube	GC-FPD	Air	3 ng mL^−1^	6 ng m^−3^	2.3	69
Sorbent tube	GC-NPD	Air	0.07 μg m^−3^	0.2 μg m^−3^	4–11	9
	GC-MS	Air	0.14 μg m^−3^	0.4 μg m^−3^	4–11	9
NTD	GC-FID	Air	0.02	0.1	3.9–6.4	Current study

The standard method of NIOSH (using sorbent tube) has different disadvantages compared to the proposed method, such as preparing a multi-stage sample and using toxic and carcinogenic solvents for analyte extraction.^11^ Also, despite the advantages such as high sensitivity and simple implementation, SPME compared to the NTD method has disadvantages such as short life, fiber fragility and limited adsorption capacity.^16,17^

**Fig. 5:**
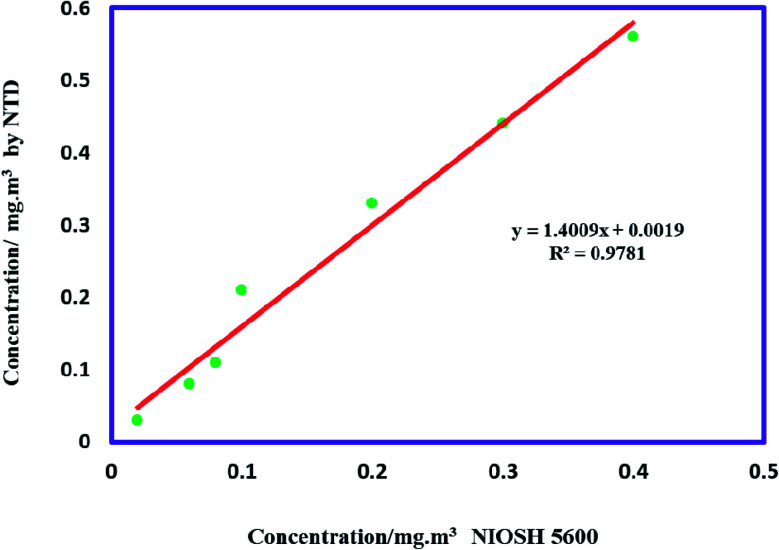
Comparison of MIP@MOF:NTD and NIOSH5600 method.

The accuracy of the proposed method was evaluated at concentrations of 0.2–3.0 times the TLV-TWA. In this way, the MIP@MOF:NTD and the NIOSH5600 methods were utilized for simultaneously sampling diazinon from the chamber. As shown in Fig. 5, there is a high correlation between of two methods (*R*^2^ = 0.9781).

### 3.7 Storage time

To evaluation of the storage time, the selected samples were analyzed immediately and 1 to 60 days after the sampling date. The storage samples at 4 °C did not show any significant difference with the controlling needles in 50 days (*P* > 0.05). Therefore, the collected samples by MIP@MOF:NTD method could be stored in the refrigerator at least for 50 days. Also, the peak area of diazinon (as a response) in NTD stored at 25 °C did not change significantly compared to the controlling values (*P* > 0.05) after 15 days. According to the standard NIOSH method (5600) recommended, the storage time of organophosphate pesticides including diazinon should be 0 and 25 °C for 10 and 29 days, respectively.^10^

### 3.8 Selectivity

To evaluate the adsorption selectivity of MIP@MOF:NTD, parathion and chlorpyriphos were selected as the potential interfering compounds due to the chemical structure similarity of parathion and chlorpyriphos to the diazinon. As illustrated in Fig. S3,† MIP@MOF:NTD provides an acceptable binding efficiency for the template molecule Compared to the NIP@MOF:NTD (non-imprinted polymer@MOF:NTD) system.

### 3.9 In-field measurements

Finally, after optimization of the effective parameters for extraction and analysis of diazinon by MIP@MOF:NTD in the laboratory, the efficiency of this method was investigated in a real environment (crop spraying). Based on the obtained results, the concentration of diazinon with the MIP@MOF:NTD method was estimated in the range of 0.008–0.01 mg m^−3^ with RSD% of 5.2–7.8%, respectively. Fig. S4† shows the obtained chromatogram of diazinon in the air by MIP@MOF:NTD and NIP@MOF:NTD (non-imprinted polymer@MOF:NTD) technique.

## 4. Conclusion

In the present study, an efficient porous adsorbent nano-composite comprising of a molecularly imprinted polymer (MIP) and a metal–organic framework (MOF) was synthesized and utilized for sampling, extraction and determination of diazinon pesticide by a needle trap device (NTD) in a laboratory and real conditions, for the first time. In the laboratory section, optimization of effective parameters on the NTD performance (such as desorption time and temperature, humidity and sampling temperature) was followed by RSM and CCD. According to the results, the optimum temperature and humidity of the sampling site were determined to be 20 °C and 25.0%, respectively. Also, the optimal values of desorption parameters (time and temperature) were 4.5 minutes and 262 °C, respectively. LOD and LOQ values were calculated to be 0.02 and 0.1 μg m^−3^, respectively, which indicated the high sensitivity of the proposed method. The developed MIP@MOF:NTD method provides an acceptable reproducibility and repeatability, high breakthrough volume, acceptable accuracy and low carryover effect. Finally, the proposed method for occupational exposure to diazinon was successfully applied in the real environment. The results exhibited that the proposed method can be successfully employed as a sensitive, solvent-free, simple, user-friendly, fast, cost-effective and environmentally friendly method for the health and environmental monitoring of diazinon in the air.

## Conflicts of interest

The authors have no conflict of interest to declare.

## Supplementary Material

RA-012-D2RA01614A-s001
