# Supplementary material for: Application of a needle trap device packed with a MIP@MOF nano-composite for efficient sampling and determination of airborne diazinon pesticide
Source: RSC Adv. 2022 May 31;12(25):16267–76. doi: 10.1039/d2ra01614a (PMC9153383; doi:10.1039/d2ra01614a)
Supplement: RA-012-D2RA01614A-s001 [file RA-012-D2RA01614A-s001.pdf]

Electronic Supplementary Material (ESI) for RSC Advances.
This journal is © The Royal Society of Chemistry 2022

Application of a Needle Trap Device packed with MIP@ MOF Nano-Composite for Efficient Sampling and Determination of Airborne Diazinon Pesticide

Razzagh Rahimpoor[1], Ali Firoozichahak *[2], Saber Alizadeh[3], Danial soleymani-ghoozhdi[4], Faeze Mehregan[5]

[1]Department of Occupational Health Engineering, Research Center for Health Sciences, School of Health, Larestan University of Medical Sciences, Larestan, Iran

[2] Department of Occupational Health, Faculty of Health, Social Determinants of Health Research Center, Gonabad University of Medical Science, Gonabad, Iran

[3]Department of Chemistry, Bu-Ali-Sina University, Hamedan, Iran.

[4] Student Research Committee, Faculty of Public Health, Kerman University of Medical Sciences, Kerman, Iran

[5] Medical student, school of medicine, Shahrekord University of Medical Sciences, Shahrekord, Iran.

**Corresponding author. Ali firoozichahak, Faculty Member, Department of Occupational Health, Faculty of Health, Gonabad University of Medical Sciences, Gonabad, Iran.*

Email:a.firoozi@edu.umsha.ac.ir*

Table S1 Domain of selected parameters and optimum conditions.

**Parameters**	**Sampling parameters**		**Desorption parameters**	
	**Sampling Temperature (°C)**	**Sampling Humidity (%)**	**Desorption Temperature(°C)**	**Desorption Time(min)**
parameter Ranges	20-60	25-70	200-290	1-6
Optimum conditions	20	25	262	4.5

Table S2 Analysis of variance (ANOVA) table for the optimization of desorption parameters of Diazinon sampled with NTD: MOF-MIP

**Source**	**Sum of Squares**	**Df**[a]	**Mean Square**	**F-Value**	**p-value Prob > F**	
Model	5.681E+006	5	1.136E+006	12.24	0.0024	significant
A-Temperature	*1.055E+006*	1	*1.055E+006*	*11.37*	*0.0119*	
B-Time	*1.053E+006*	1	*1.053E+006*	*11.35*	*0.0119*	
AB	*11772.25*	1	*11772.25*	*0.13*	*0.7322*	
A^2	*9.921E+005*	1	*9.921E+005*	*10.69*	*0.0137*	
B^2	*1.215E+006*	1	*1.215E+006*	*13.09*	*0.0085*	
Residual	6.497E+005	7	92812.42			
Lack of Fit	*2.987E+005*	3	*99566.72*	*1.13*	*0.4356*	not significant
Pure Error	*3.510E+005*	4	*87746.70*			
Cor Total	6.331E+006	12				
R-Squared	0.8974					
Adj R-Squared	0.8241					

[a] df: Degree of freedom

Table S3 Analysis of variance (ANOVA) table for the optimization of sampling parameters of Diazinon sampled with NTD: MOF-MIP

**Source**	**Sum of Squares**	**Df**[a]	**Mean Square**	**F-Value**	**p-value Prob > F**	
Model	6.161E+006	5	1.232E+006	55.75	< 0.0001	significant
A-Temperature	*2.685E+006*	*1*	*2.685E+006*	*121.48*	*< 0.0001*	
B-Humidity	*2.021E+006*	*1*	*2.021E+006*	*91.42*	*< 0.0001*	
AB	*7.841E+005*	*1*	*7.841E+005*	*35.47*	*0.0006*	
A^2	*4.641E+005*	*1*	*4.641E+005*	*21.00*	*0.0025*	
B^2	*25949.82*	*1*	*25949.82*	*1.17*	*0.3145*	
Residual	1.547E+005	7	22104.87			
Lack of Fit	*52456.92*	*3*	*17485.64*	*0.68*	*0.6069*	not significant
Pure Error	*1.023E+005*	*4*	*25569.30*			
Cor Total	6.316E+006	12				
R-Squared	0.9755					
Adj R-Squared	0.9580					

[a] df: Degree of freedom

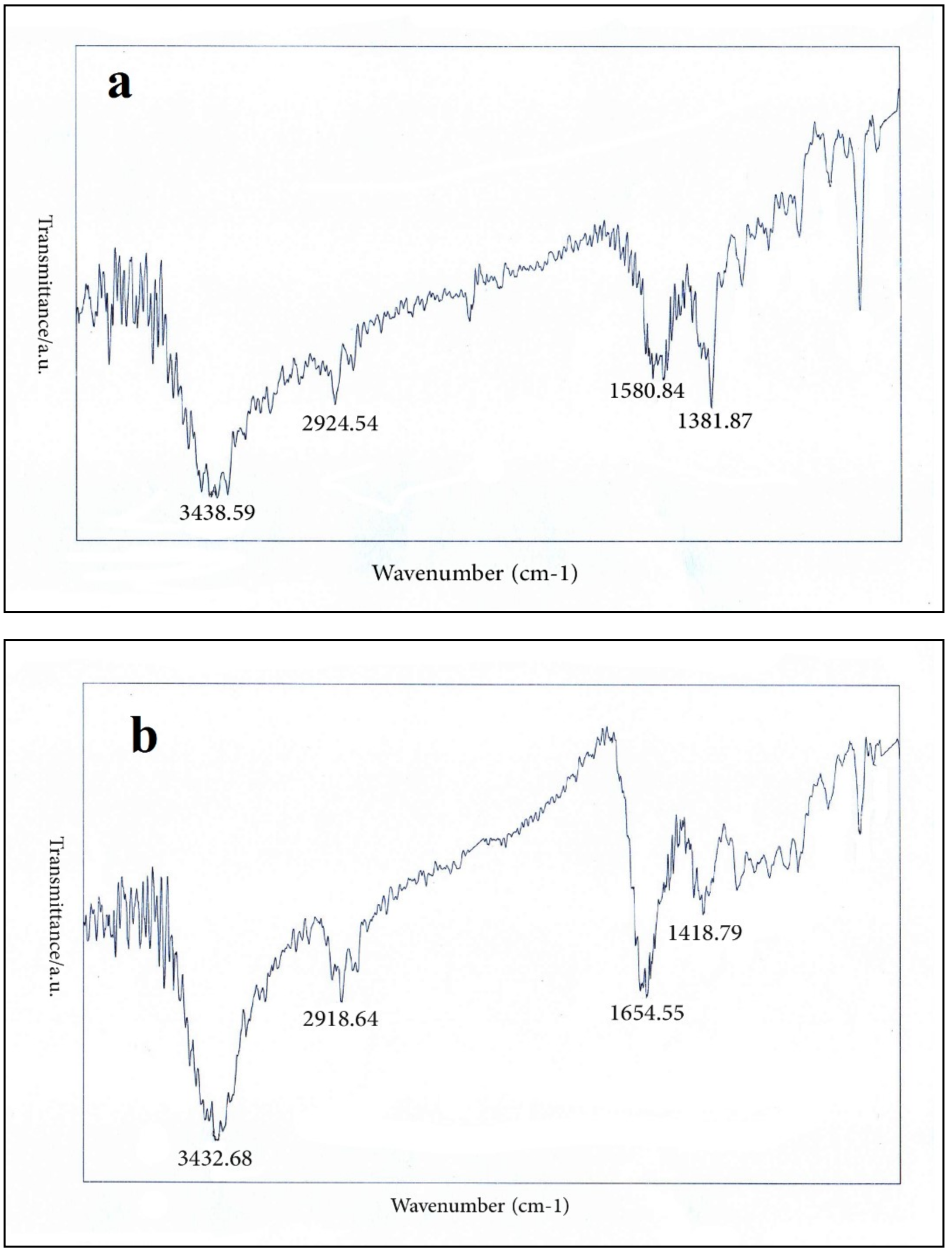

Fig. S1: FT-IR spectra of MOF and (a) and MIP@MOF (b)

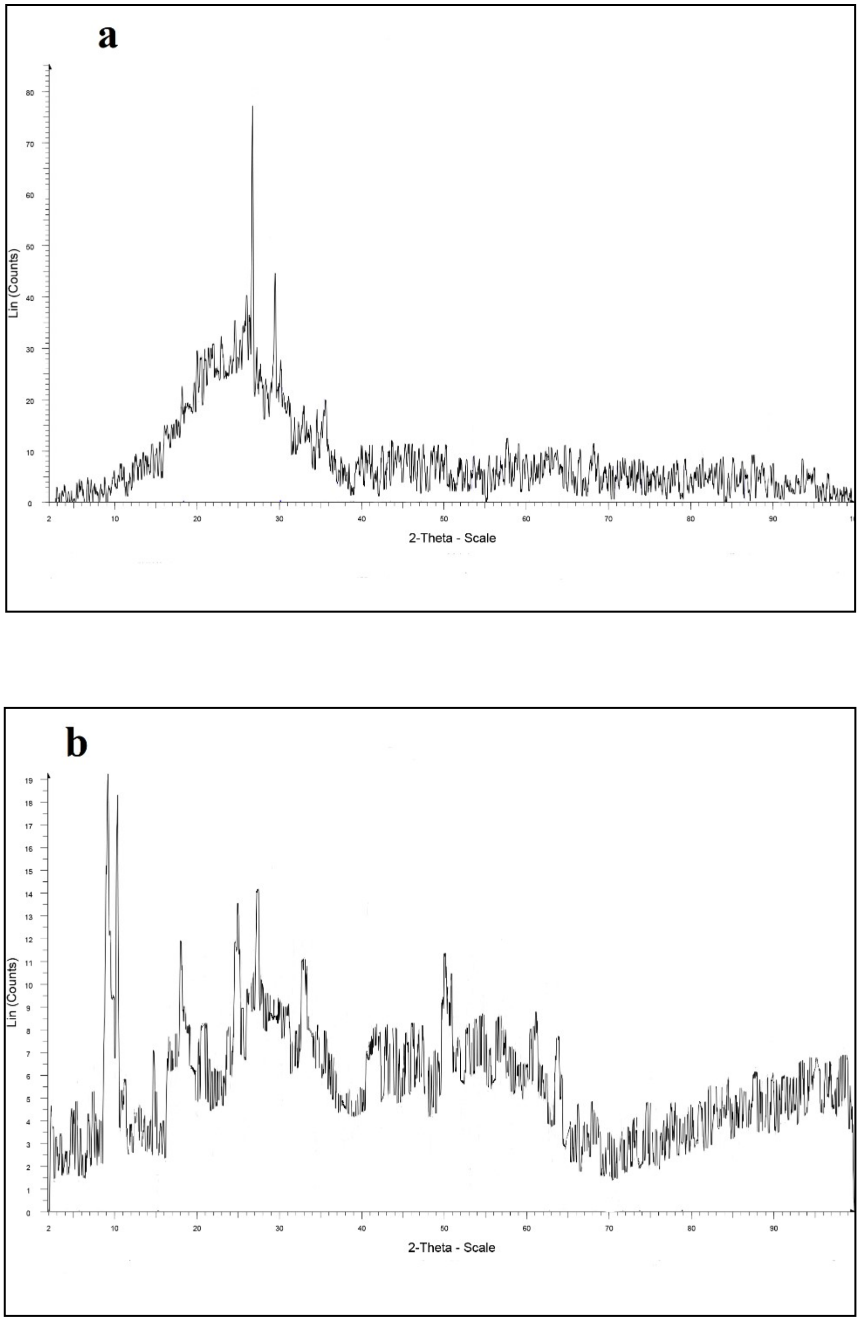

Fig. S2: The XRD patterns of MIP (a) and MIP@MIL-101(Fe) (b)

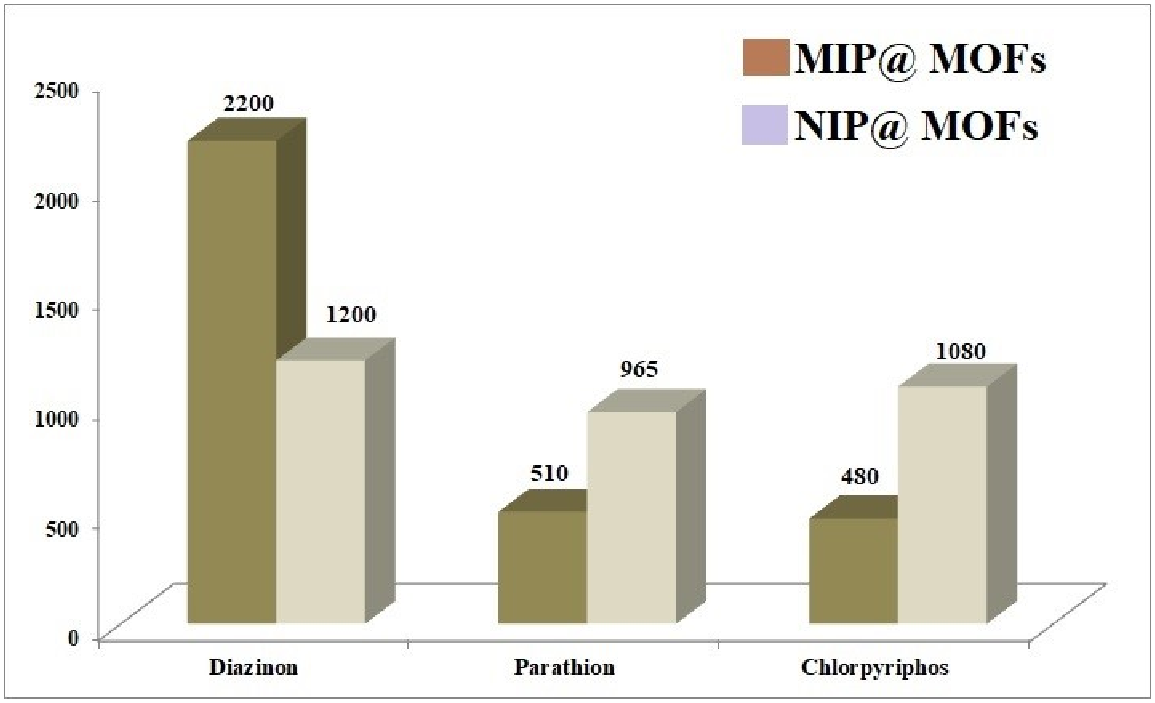

Fig. S3: Selectivity of MIP@MOF and NIP@MOF techniques for the sampling, extraction and determination of diazinon from the air sample

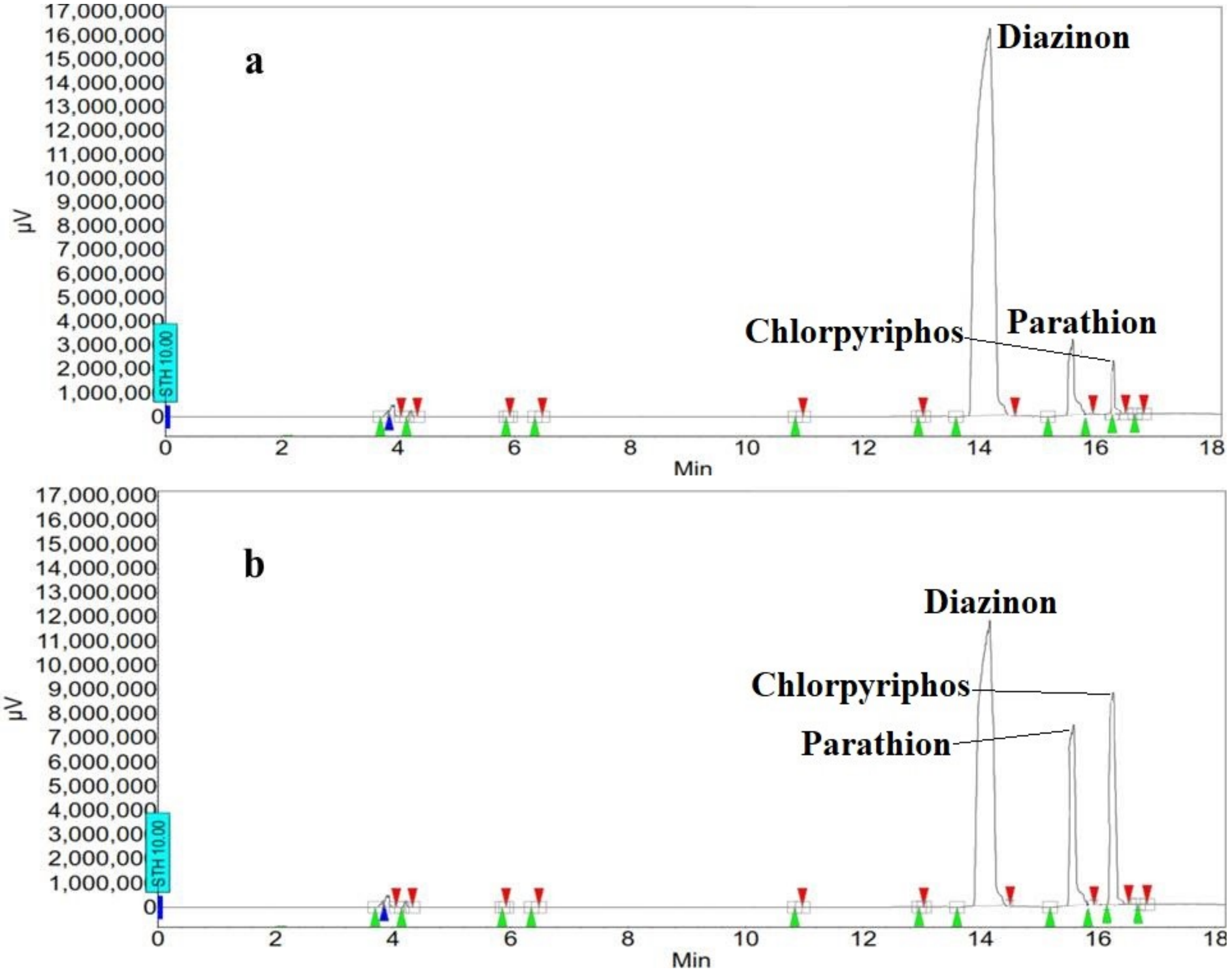

Fig. S4: The obtained chromatograms from the extraction of diazinon in the air by (a) MIP@MOF:NTD and (b) NIP@MOF:NTD systems
